# Research progress on drugs targeting the TGF-*β* signaling pathway in fibrotic diseases

**DOI:** 10.1007/s12026-022-09267-y

**Published:** 2022-02-11

**Authors:** Ning Shi, Zhihong Wang, Hecheng Zhu, Weidong Liu, Ming Zhao, Xingjun Jiang, Jin Zhao, Caiping Ren, Yan Zhang, Longlong Luo

**Affiliations:** 1 Department of Neurosurgery, Cancer Research Institute, School of Basic Medical Science, Xiangya Hospital, Central South University, Changsha, 410008 China; 2 The Key Laboratory of Carcinogenesis of the Chinese Ministry of Health and the Key Laboratory of Carcinogenesis and Cancer Invasion of the Chinese Ministry of Education, Central South University, Changsha, 410008 China; 3 State Key Laboratory of Toxicology and Medical Countermeasures, Beijing Institute of Pharmacology and Toxicology, Taiping Road #27, Beijing, 100850 China; 4 Changsha Kexin Cancer Hospital, Changsha, 410205 Hunan China; 5 Department of Obstetrics and Gynecology, First Medical Center, General Hospital of Chinese PLA, Beijing, China

**Keywords:** TGF-β, TGF-β antibodies, TGF-β signaling pathways, Fibrosis disease

## Abstract

Tissue fibrosis is a key factor leading to disability and death worldwide; however, thus far, there are no approved treatments for fibrosis. Transforming growth factor (TGF)-β is a major pro-fibrotic cytokine, which is expected to become a target in the treatment of fibrosis; however, since TGF-β has a wide range of biological functions involving a variety of biological processes in the body, a slight change in TGF-β may have a systematic effect. Indiscriminate inhibition of TGF-β can lead to adverse reactions, which can affect the efficacy of treatment. Therefore, it has become very important to explore how both the TGF-β signaling pathway is inhibited and the safe and efficient TGF-β small molecule inhibitors or neutralizing antibodies are designed in the treatment of fibrotic diseases. In this review, we mainly discuss the key role of the TGF-β signaling pathway in fibrotic diseases, as well as the development of fibrotic drugs in recent years, and explore potential targets in the treatment of fibrotic diseases in order to guide subsequent drug development.

## Introduction

According to relevant statistics, in the USA, nearly 45% of deaths for patients who suffered from various diseases can be attributed to tissue fibroproliferative diseases [1]. Thus far, there is no approved treatment effective against fibrosis. Although the average life expectancy has been greatly improved as medical technology continues to develop, there is an increasing incidence of organ fibrotic diseases among younger patients and more generalized disease patterns [2–6]. Therefore, there is an urgent need to develop effective drugs for the treatment of fibrosis.

There are many factors leading to fibrosis, including occupation, hereditary disease, lifestyle, aging [7, 8], as well as physical, chemical, and biological factors, etc. [6, 9, 10]. For example, silicosis is a serious occupational disease in China. Due to the long-term exposure to the environment containing silica dust, these particles will accumulate in the lungs and will not be metabolized by the body. Workers who work for a long time will form nodules in the lungs, forming inflammatory lesions and inducing pulmonary fibrosis, and eventually causing irreversible damage to the body [11, 12]. In addition to the above reasons, studies have also reported that the lack of neuraminidase 1(NEU1) is closely related to the formation of fibrosis in muscles, kidneys, liver, heart, and lungs[13]. There is also a genetic link between rheumatoid arthritis-related interstitial lung disease and idiopathic pulmonary fibrosis [14, 15]. Finally, a poor lifestyle is also the main cause of chronic inflammation and even organ fibrosis.

**Fig. 1:**
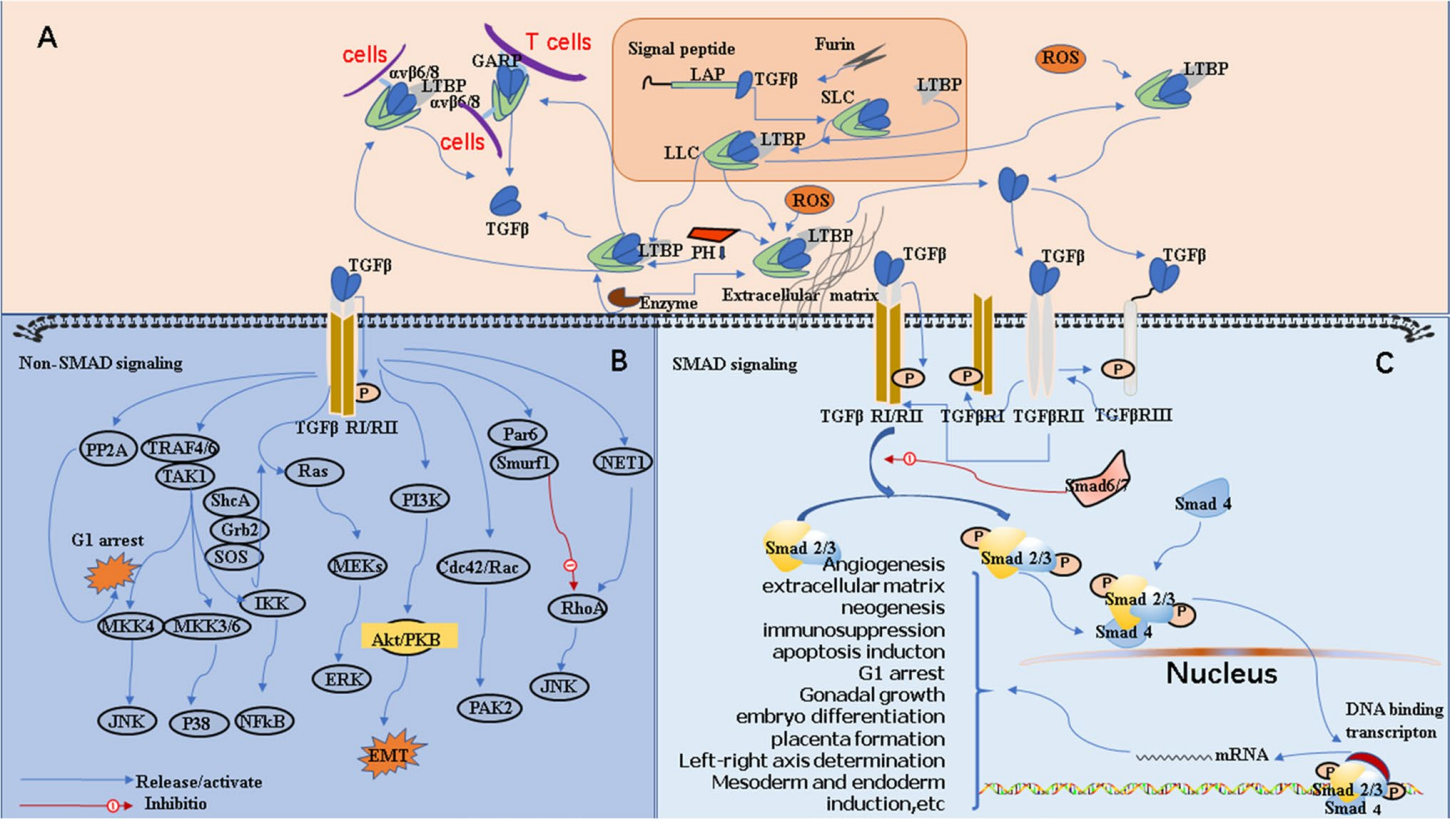
Schematic diagram of TGF-β signaling pathway. **A** Synthesis of TGF-β precursor and activation of mature TGF β. In the cytoplasm, SLC and LTBP combine to form a large latent complex (LLC) that is secreted into the peripheral circulation of the cells. LTBP can mediate the non-covalent binding of LLC to fibrillin and promote the release of TGF-β by different means such as proteases, integrins, pH, and reactive oxygen species-mediated ways as mentioned above. **B** Nonclassical Smad signaling pathway mediated by TGF-β. TGF-β phosphorylates downstream adaptor molecules such as RhoA, Ras, TAK1, and P13K and activates the downstream signal amplification cascade including MKKs and MEKs, JNK/SPAK, p38, and other pathways. **C** Classical Smad signaling pathway mediated by TGF-β. TβRIII presents TGF-β to TβRII, and TβRII combined with TGF-β recruits and phosphorylates TβRI. Finally, the dimerized TβRI and the dimerized TβRII are cross-linked and then trigger the intracellular TGF-β signaling pathway

In the process of fibrosis formation, a variety of molecular mechanisms and changes in the microenvironment are involved. For example, TGF-β, platelet-derived growth factor (PDGF), connective tissue growth factor (CTGF), interleukin-4 (IL-4), interleukin-13 (IL-13), and other cytokineshave chemotactic effects on fibroblasts and regulate the synthesis and degradation of collagen, which in turn is closely related to the occurrence and development of fibrosis [16–19]. In addition, oxidative stress and inflammation can promote the formation of fibrosis [18–21]. Among the above factors that cause fibrosis, TGF-β plays a vital role. As shown in Fig. 1, TGF-β will be overactivated in persistent inflammation such as tissue damage. The activated TGF-β not only mediates SMAD signaling pathway but also activates the PI3K-AKT-mTOR signaling pathway for transcriptional regulation, which in turn promotes epithelial-mesenchymal transition (EMT) and triggers the accumulation of extracellular matrix [22, 23]. On the other hand, TGF-β can also regulate the activity of RhoA, promoting the activation of Rho-related kinase (ROCK) and inhibiting cofilin (CFL) [24]. Rac and Cdc42 are also involved in the non-Smad signaling pathway mediated by TGF-β signaling [25–29]. Most notably, TGF-β1 is the strongest profibrotic cytokine discovered to date [30–34]; many studies have shown that blocking the TGF-β1 pathway can improve organ fibrosis [32, 35]. However, TGF-β participates in the regulation of a variety of other signal pathways in the body and has an important role in maintaining physiological homeostasis (including immune regulation and tumor suppression) [32]. Therefore, in the drug design process, it is necessary to consider minimizing the potential adverse effects of systemic blocking of TGF-β.

Judging from the current clinical incidence rate of fibrotic diseases, pulmonary fibrosis, renal fibrosis, and liver fibrosis are the most common fibrotic diseases, and there is an enormous need for the treatment of these diseases clinically which is not being met [36–38]. An understanding of fibrotic disease mechanisms has accelerated the research and development of its clinical treatment. Currently, dozens of anti-fibrosis drugs with different targets are under development. The main treatments are listed below. (1) Chemically synthesized oligonucleotides as miRNA inhibitors or analogs. Since studies have shown that some miRNAs could upregulate or downregulate the transcription of specific genes related to fibrosis [39–43]. For example, it was found that the injection of miR-326 into mice with bleomycin-induced pulmonary fibrosis caused significant downregulation of TGF-β1, Smad3, matrix metalloproteinase-9 (MMP-9), and upregulation of Smad7, which in turn have good anti-fibrosis effects [39]. (2) Recombinant serum amyloid P (Pentraxin-2), which has the function of regulating the natural immune response, has the function of inhibiting the differentiation of monocytes into fibroblasts or fibrosis-promoting phenotypes and activating macrophage subpopulations. It is located at the injury site and has a dual effect by inhibiting fibrosis and promoting repair. PRM-151 is a recombinant protein of Pentraxin-2, which has shown good efficacy in the treatment of pulmonary fibrosis and is currently undergoing phase III clinical trials in patients with idiopathic pulmonary fibrosis (IPF) [22, 44]. (3) Another potential therapeutic target is LoxL2 (a lysyl oxidase family member), which plays a key role in ECM cross-linking, affecting the expression of certain specific genes, which are related to fibrosis and carcinogenesis [45–50]. GS-6624 is a humanized IgG4 monoclonal antibody that targets LoxL2, blocks the activity of LoxL2, reduces the production of ECM by myofibroblasts, and has shown good efficacy in treating fibrosis [51]. (4) Drugs that target IL-4 and IL-13; IL-4 and IL-13 are also potential targets in the treatment of fibrosis, which are important for mediating innate immune activation and helper T-cell 2 (Th-2) cell response[16]. Studies have shown that IL-13 participates in the generation of TGF-β1 by regulating IL-13 Ralpha2 receptors and promotes the process of fibrosis [52–54]. (5) Drugs that target TGF-β [33, 55, 56]. For example, it has been found that the small molecular inhibitor LY364947 selectively inhibits TGF-β1 could effectively block the activation and proliferation of mouse cardiac fibroblasts with fewer side effects and is safer than pan-TGF-β blocking in vivo [33]. In order to avoid harmful effects, choosing appropriate inhibitory strength, drug duration, combination with other drugs that can reduce side effects [57], and local inhibition of TGF-β1 signaling may be good drug development strategies. For example, designing bifunctional antibodies to make the drug more enriched at the lesion site may be a good choice. Since TGF-β has a high degree of pleiotropy, there are still many problems to be resolved in fibrosis treatment by targeting TGF-β. However, it is clear that this cytokine has great therapeutic value. In this paper, we will outline the application and potential of targeting TGF-β in the treatment of tissue fibrosis by introducing advances made in scientific research involving mechanisms that TGF-β may be involved in.

## TGF-β superfamily signaling pathway

### TGF-β superfamily members

TGF-β superfamily members can regulate the proliferation, differentiation, apoptosis, adhesion, and migration of a variety of cells, such as macrophages, T cells, B cells, immature hematopoietic cells, neutrophils, and dendritic cells, etc. [58–61]. The TGF-β family consists of 33 members, including TGF-β, growth differentiation factors (GDFs), bone morphogenetic protein (BMP), activin, NODAL, and anti-Mullerian hormone (AMH) [53, 62].

TGF-β has three subtypes in mammals, namely TGF-β1, TGF-β2, and TGF-β3. Although their structures are highly similar, they perform different functions. TGF-β1 plays an important role in maintaining the stability of the body. It can activate fibroblasts and promote the synthesis of the extracellular matrix [63]. Studies have also shown that loss of TGF-β1 may cause defects in hematopoietic and endothelial cell differentiation or autoimmune diseases [64], and lack of TGF-β2 can affect epithelial-mesenchymal interactions, cell growth, extracellular matrix production, and result in tissue remodeling disorders, causing defects in the heart, lungs, cranium and face, limbs, spine, eyes, inner ears, and urogenital system [65]. TGF-β3 plays an important role in the normal morphogenesis of the palate and lungs and participates in epithelial-mesenchymal interaction [66], in which it can reduce scar formation during wound healing [67]. Among the three TGF-β subtypes, TGF-β1 is the most fully studied member of the transforming growth factor family, which plays a major role in tumor development and tissue fibrosis.

### TGF-β receptors

TGF-β receptors are divided into three categories: transforming growth factor-β type I receptor (TβRI), transforming growth factor-β type II receptor (TβRII), and transforming growth factor-β type III receptor (TβRIII)[68, 69]. TβRII contains a serine/threonine-rich sequence, which can undergo autophosphorylation. TβRI contains a conservative serine/glycine-rich sequence (TTSGSGSGLP, also known as GS region), which plays a key part in TβRI activation. Both TβRI and TβRII can directly participate in the signal transmission process [69]. TβRIII is a co-receptor of the TGF-β superfamily. The extracellular domain of TβRIII contains an independent amino terminal domain, a zona pelucida domain (ZPD) potentially involved in the oligomerization reaction of the receptor, and two independent TGF-β ligand-binding domains [70]. TβRIII can mediate both the classic Smad signaling pathway and the non-classical Smad signaling pathway to regulate the downstream signaling of TGF-β [71–73] and plays a role in ligand presentation in the TGF-β classic signaling pathway. However, in many diseases, TβRIII can block the TGF-β signaling pathway by forming a complex with TβRI and TβRII [74]. In the classical TGF-β pathway, active TGF-β can first bind to TβRIII, then be presented to the receptor complex composed of TβRI and TβRII, and initiate downstream signaling pathways (Fig. 1) [75, 76].

### TGF-β secretion and activation

The TGF-β family members are composed of latency-related peptide (LAP), precursor domain, and C-terminal TGF-β fragment, where the precursor domain and growth factor are connected by a cleavage site of proprotein convertase (PC) [77, 78]. The TGF-β precursor is first synthesized on the rough endoplasmic reticulum [79]. After being transferred into the Golgi complex and cleaved by the invertase furin, it is bound with LAP homodimer in a non-covalent form and forms a symmetrical heterotetrameric structure, which is called small latent complex (SLC) [79]. This SLC functions as a cover to prevent mature TGF-β from binding to cell surface receptors. SLC can be hydrolyzed by protease or non-protease to form mature TGF-β with biological activity [80, 81]. During the process, the homodimer part of LAP is connected by two disulfide bonds, and the interaction of the mature TGF-β part is also stabilized by disulfide bonds [82].

The activation of latent TGF-β complex involves several important molecular mechanisms as shown in Fig. 1. (1) The arginine-glycine-aspartate (RGD) motif in TGF-β precursor fragment bound with integrin β chain via non-covalent bonds. When integrin αvβ6/αvβ8 on stromal cells binds to RGD sequence on the TGF-β precursor domain, under the combined action of mechanical force of cytoskeleton and the reaction force of the extracellular matrix or cells presenting TGF-β, the closed loop of precursor domain opens and mature TGF-β with biological function is released [79, 81, 83]. (2) The TGF-β precursor fragment is cleaved by proteases such as metalloprotease MMP-2, MMP-3, MMP-9, and plasmin to release mature TGF-β [79, 84]. (3) Reactive oxygen species (ROS); in the process of liver fibrosis, activated hepatic stellate cells release a large amount of ROS, and a high level of ROS can promote the release of transforming growth factor-β [85]. (4) Changes in pH; in a tissue microenvironment with a low pH, mature TGF-β is released more easily [54, 86, 87]. (5) Studies have shown that the small latent complex can also bind to glycoprotein-A repetitions predominant (GARP, usually overexpressed on the membrane of the Treg cells) on cell surfaces, leading to the release of active TGF-β and the regulation of the proliferation and differentiation of Treg cells [80, 83] (Fig. 1).

### TGF-β signaling pathway

Once activated, mature TGF-β initiates transmembrane signaling by binding itself to two distinct transmembranes Ser/Thr protein kinases, termed as TβRI and TβRII receptors. Among them, transforming growth factor-β can also bind to the helper receptor TβRIII, and then the signal molecules are transmitted to TβRI and TβRII by T-β RIII. TβRII can phosphorylate specific cytoplasmic GS domains of TβRI, leading to conformational regulation of TβRI so as to phosphorylate Smad2 and Smad3 proteins. Subsequently, phosphorylated Smad2 and Smad3 proteins bind to Smad4 protein to form heterologous complexes, with the formed heterotrimers translocating into the nucleus and bound to DNA to regulate the transcription of multiple target genes [62, 88–90]. In the nucleus, with the participation of certain DNA-binding proteins, Smad molecules act on specific target genes to regulate their expression. For example, phosphorylated Smad2/3/4 heterocomplexes can form complexes with p300 and CREB-binding proteins (CBP) to promote the transcription of target genes such as plasminogen activator inhibitor-1 (PAI-1), collagen type I alpha 1 (COL1A1), and connective tissue growth factor (CTGF) [91] to regulate the production of the extracellular matrix. In addition to interacting with transcription factors, Smad complexes have been shown to regulate epigenetic modifications by transferring histone acetyl and methyltransferases to specific gene sites. Phosphorylated Smad2/3 protein can bind to M6A methyltransferase complex to regulate m6A RNA methylation modification of target genes, thus exerting the biological function of DNA repair and posttranscriptional regulation [62, 92].

More and more pieces of evidence clearly demonstrate that in addition to activating classic Smad-dependent signaling, TGF-β also participates in nonclassical signaling pathways, which mainly include MAP kinases, PI-3 kinase-Akt, and Rho-like GTPase [93–96]. Figure 1 depicts the classic TGF-β signaling pathway and nonclassical TGF-β signaling pathway.

## The role of TGF-β in the progression of tissue fibrosis

**Fig. 2:**
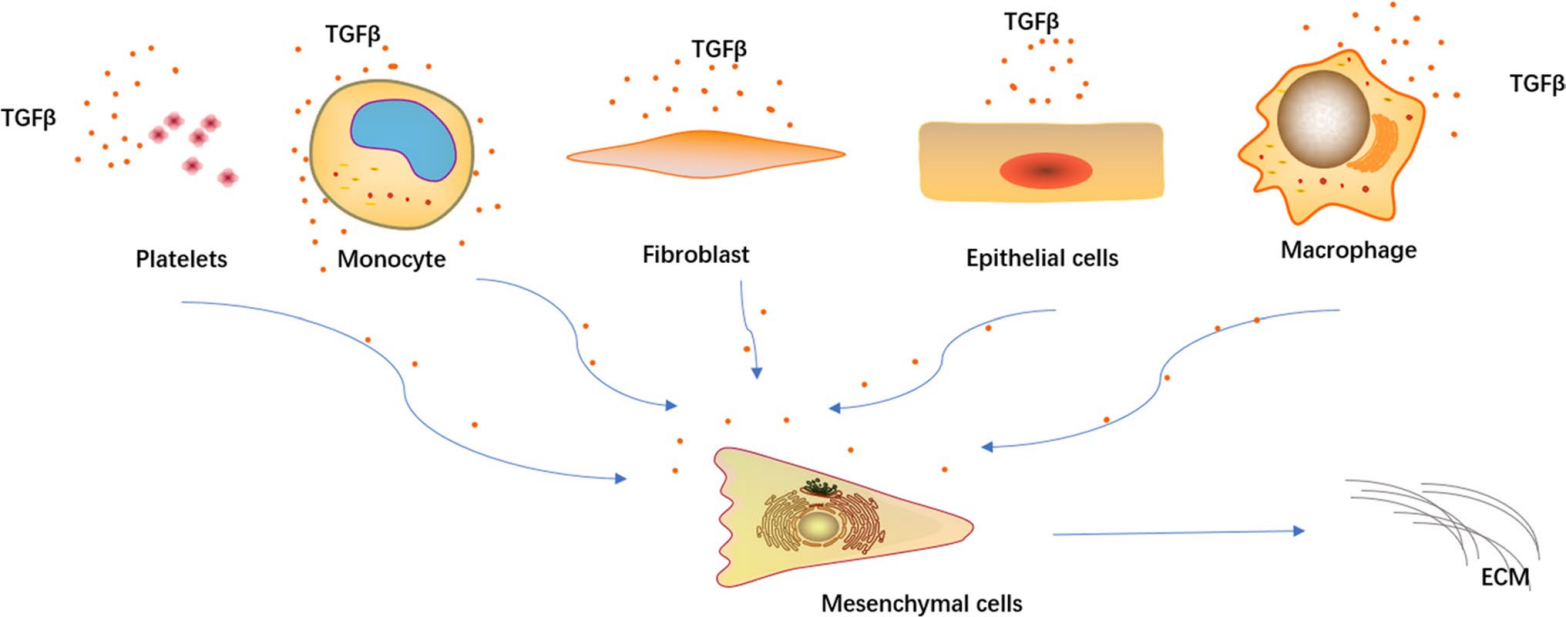
Schematic diagram of the partial sources of TGF-β. Under the conditions of organ or tissue damage, TGF-β can increase its own expression by inducing the secretion of TGF-β from epithelial cells, immune cells, fibroblasts, etc., thereby promoting the excessive production of extracellular matrix

It has been well known that fibrosis is not a disease but a result of tissue damage repair dysfunction [54]. TGF-β plays an important role in this process. During tissue damage repair, mesenchymal cells undergo significant metabolic changes to promote energy-consuming cell functions including cell proliferation and protein synthesis [97–99]. The increased glycolysis activity of fibroblasts results in the synthesis of pyruvic acid and lactic acid. The generated lactic acid reduces extracellular pH, which induces the activation of potential TGF-β1 [54, 100]. Meanwhile, integrin-mediated TGF-β activation promotes the expression of IL-17A, which increases the expression of TGF-β receptors in fibroblasts, thereby promoting the response of fibroblasts to TGF-β signals [54, 101–103]. In addition, in fibroblasts stimulated by TGF-β1, the increase in the level of glutaminase promotes the decomposition of glutamine, endows the cells with antiapoptotic properties [104], finally promotes the production and stability of collagen through mTOR signal transduction [105]. Studies have shown that inhibiting glutaminase I can improve the symptoms of pulmonary fibrosis induced by bleomycin and TGF-β1 in vivo [106]. And TGF-β is one of the main regulators of cell differentiation, migration, proliferation, and gene expression [107]. In injured or diseased tissues, sustained, dysregulated, or hyperactive TGF-β transcriptional activation leads to enhanced fibrogenesis, which impairs normal tissue regeneration and may cause dysfunction by interfering with the structure of organ structural units [108]. The specific cell source of TGF-β in the body is unclear. Epithelial cells, platelets, T cells, fibroblasts, and mast cells can all produce TGF-β [109–112]. In the study of acute and chronic fibrotic injury models, it was found that disrupting the pathways involved in the recruitment of macrophages can reduce the synthesis of TGF-β and relieve fibrosis, which indicates that macrophages are also an important source of TGF-β [113, 114]. The continuous increase in TGF-β will further deepen the degree of fibrosis and form a serious vicious circle (Fig. 2).

## Anti-fibrosis drug research

Fibrosis is a common manifestation of chronic tissue damage. A further understanding of the mechanisms of fibrotic diseases will help accelerate the development of its clinical treatment. At present, the main mechanism of action used by drugs under development against fibrosis is related to the inhibition of various factors in the formation of fibrosis, including inhibition of the signaling of cytokines such as TGF-β, PDGF, and CTGF, inhibition of fibroblast division and proliferation, regulation of collagen synthesis and degradation, and regulation of oxidative stress, inflammation, and other reaction processes that contribute to the formation of fibrosis [17, 38, 54, 115]. For example, HSC activation is a key step in the formation of liver fibrosis, so HSC is regarded as an important target in the development of drugs used against liver fibrosis [44]. Since the discovery of the importance of TGF-β in fibrotic diseases, drug research targeting the TGF-β signaling pathway has increased significantly [58, 67, 90]. Related drug types include antisense oligonucleotides (AON), neutralizing antibodies, cyclic RGD pentapeptides, TGF-β ligand traps, and small molecule kinase inhibitors (SKIs), etc.[80, 116–118]. In early anti-fibrosis drug research, researchers found that pan-TGF-β antibody drugs might cause cardiotoxicity [54, 119]. Through the mouse model experiments, it was found that the cardiotoxicity caused by pan-TGF-β antibody drugs may be related to the indiscriminate blocking of TGF-β2 and TGF-β3 signaling pathways [119], the results also provide guidance for the design of selective targeting of TGF-β drugs. There are already a variety of drugs targeting TGF-β superfamily members or their receptors under development, such as the highly selective antibody SRK-181 developed by Scholar Rock, which targets the TGF-β1 precursor. The main mechanism of SRK-181 is to prevent the cleavage of TGF-β1 precursor and release mature TGF-β by binding to TGF-β1 precursor [120]. In the 4-week repeated-dose rat toxicity study of SRK-181 (the highest dose was 100 mg/kg, which was much higher than the dose required to induce a strong antitumor response in combination with PD-1 antibody), the researchers did not observe other histological features of cardiac valvulopathy or cardiotoxicity. This indicates that selective blocking of TGF-β1 activation may avoid the dose-limiting toxicity caused by the indiscriminate TGF-β inhibitor drugs to a certain extent [119, 121]. The AVID200 developed by Forbius has also attracted widespread attention [122, 123]. AVID200 is a highly effective and selective inhibitor specifically targeting TGFβRII mutant that enhances the binding activity of TGFβRII to TGF-β1 and TGF-β3 and thus greatly reduces the binding activity to TGF-β2 [123]. In clinical phase I trials, researchers found that AVID200 has a good anti-fibrosis effect, and preclinical models showed that blocking TGF-β signal transduction can reverse myelofibrosis and restore hematopoietic function without safety risks [122–124]. Other than that, a polypeptide drug HTPEP-001 targeting TGF-β1 for the treatment of IPF, which was developed by Chengdu Huitai Biotechnology has also shown good results in preclinical experiments [125]. The study found that by inhibiting the production of active TGF-β1 and Smad signaling, aerosol inhalation of HTPEP-001 effectively blocked the fibrosis process in the rat model of bleomycin-induced pulmonary fibrosis, and there were no obvious adverse events related to immunological or histological changes [125]. The above studies all support selective targeting TGF-β as a promising direction for the treatment of fibrotic diseases. The table below summarizes some of the drugs under development for the treatment of fibrotic diseases (Table 1).

**Table 1:** Summary of some drugs under development for fibrosis

Drug	Disease	Target	Phase	Identifier	Supplement
Drugs targeting TGF-β signaling pathway for fibrosis treatment					
Pirfenidone	Pulmonary fibrosis	TGF-β	Phase II/III already listed	NCT04461587 NCT04607928 NCT02958917 NCT02161952 NCT01366209	Broad-spectrum anti-fibrosis drugs
NIS793	Myelofibrosis	TGF-β1	Phase II	NCT04097821	Single drug or combined with PDR001, MBG453, or other drugs to treat myelofibrosis
Fluorofenidone	Liver fibrosis	TGF-β	Phase I	2016L09979	Same target as pirfenidone
KER-050	Myelofibrosis	TGF-β	Phase II	NCT04419649	KER-050 works by inhibiting TGF-β signaling and is being developed for the treatment of cell tumors, myelodysplastic syndrome, and myelofibrosis
AVID200	Myelofibrosis	TGF-β1/3	Phase I	NCT03895112	It was found in preclinical models that blocking TGF-β signal transduction can reverse myelofibrosis and restore hematopoietic function
P144	Skin fibrosis	TGF-β1	Phase II	NCT00781053 NCT00574613	TGF-β1 inhibitors are used to block the interaction between TGF-β1 and TGF-βRIII receptors, thereby blocking its biological effects
HTPEP-001	Pulmonary fibrosis	TGF-β1	Preclinical experiment		HTPEP-001 can effectively block the fibrosis process of the rat lung fibrosis induced by bleomycin
SB-431542	Fibrosis	TGF-βRI	Preclinical experiment		Can inhibit the kinase activity of ALK5 (IC50 = 94 nmol·L-1) and ALK4 and ALK7 in vitro but has no effect on ERK, JNK, and p38 MAPK signaling pathways
A83-01	Fibrosis	TGF-βRI	Preclinical experiment		A83-01 is a selective small-molecule inhibitor that inhibits TGF-β type I receptors
IN-1130	Renal fibrosis	TGF-βRI	Preclinical experiment		In the rat renal fibrosis model, IN-1130 can reduce the expression levels of phosphorylated Smad2, fibronectin, α-SMA, and type I collagen, significantly inhibiting the process of renal fibrosis
STX-100	Idiopathic pulmonary fibrosis	αVβ6	Phase II	NCT01371305	STX-100 cannot inhibit inactive TGFβ but selectively inhibits activated TGF-β in pathological tissues
PLN-74809	Idiopathic pulmonary fibrosis	αVβ1/αVβ6	Phase II	NCT04396756	By inhibiting integrins, it can specifically antagonize the TGF-β signaling pathway in fibrotic tissues
Drugs targeting other pathways for fibrosis treatment					
Sorafenib	Liver fibrosis	VEGFR2/PDGF-β	Phase III	NCT01849588	Can significantly improve liver damage and liver fibrosis and promote angiogenesis
Imatinib	Liver fibrosis	PDGFR	Phase I	NCT00025415	Can significantly reduce the proliferation of HSC in the short term. Effective in the early stages of liver fibrosis
BMS-986036	Nonalcoholic steatohepatitis/liver fibrosis	FGF21 analogs	Phase II	NCT02413372	Has mild adverse reactions, such as diarrhea, nausea, and frequent bowel movements
Hydronidone	Liver fibrosis	FGFR1	Phase II	NCT02499562	Food intake will reduce the absorption rate of hydronidone
Simvastatin	Liver fibrosis	HMG-CoA reductase	Already listed		Representative statin drugs
VBY-376	Nonalcoholic steatohepatitis/fibrosis	Cathepsin B	Phase I	NCT00557583	Inhibit the activity of cathepsin B. Can slow down or inhibit the proliferation of HSC
Obeticholic acid	Liver fibrosis	FXR	Phase III	NCT02548351 NCT03979417	Significant improvement in fibrosis. Only a few patients have mild adverse reactions such as itching
PRM-151	Pulmonary fibrosis/myelofibrosis/liver fibrosis, etc	Pentraxin-2	Phase III	NCT04594707 NCT04552899	PRM-151 can activate macrophages and reduce tissue fibrosis
GS-6624	Pulmonary fibrosis	LoxL2 monoclonal antibody	Phase II	NCT01759511 NCT01672879	By binding to lysyl oxidase, it has an immunomodulatory effect
Liraglutide	Liver Fibrosis	GLP-1	Phase III	NCT02654665	GLP-1 can increase insulin release and reduce glucagon secretion, reduce liver steatosis, and improve liver fibrosis

It is well known that the tissues surrounding the tumor microenvironment are rich in dense fibrotic cells, which are usually referred to as cancer-associated fibroblasts (CAF) [126]. Therefore, the occurrence and development of tumors are closely related to fibrosis. The progress of drugs targeting TGF-β for tumor therapy under development is also summarized in Table 1. More and more drugs targeting TGF-β are being developed to treat diseases such as fibrosis and tumors, which expand our understanding of the TGF-β signaling pathway and its mechanism of action and further promote the development of new anti-fibrotic drugs targeting TGF-β with few or no side effects.

## Conclusions

With increasing in-depth research on the pathogenesis of fibrotic diseases, it has been shown that the TGF-β signaling pathway is closely related to organ fibrosis [54, 57, 67, 83, 91]. Since TGF-β participates in the regulation of multiple signaling pathways in the body, and TGF-β is closely related to the body’s metabolism, aging, circadian rhythm, epigenetics, EMT, and other cellular processes, learning how to regulate the TGF-β signaling pathway for the treatment of fibrosis while avoiding toxic side effects has become key to drug development [68, 127–129]. At present, by targeting different action sites of TGF-β and receptors, the search, design, and screening of various efficient and low-toxic novel small molecule inhibitors have become a research hotspot. Although some preclinical and clinical drug candidates for blocking TGF-β signaling pathway exhibit some side effects, such as pirfenidone (drug details are shown in Table 1), its anti-fibrosis effect is still quite encouraging [130], which provides some enlightenment for the development of new inhibitors of the TGF-β signaling pathway. However, a single drug often leads to obvious adverse reactions due to factors such as a single target and a large dose; hence, combination therapy has become the treatment trend of fibrosis disease. In addition, the use of the abovementioned inhibitors targeting the TGF-β signaling pathway reasonably combined with other drugs of different action mechanisms to treat a variety of fibrosis-related diseases has also attracted the attention of many researchers. In short, with the continuous improvement of drug development strategies and the increasing number of safe and effective small molecule inhibitors, it is believed that more effective drugs targeting TGF-β signaling for the treatment of fibrosis will enter the clinical practice.

Since a group of abnormal proliferation cells with high fibrotic characteristics also exists in most tumor microenvironments in addition to being a popular target for fibrotic diseases, TGF-β signaling is also one of the most popular targets in the field of tumor immunotherapy in recent years [97]. The bifunctional antibody drug M7824, which is being developed by Merck, has attracted widespread attention in the pharmaceutical industry due to its good synergistic therapeutic mechanism for tumors [131, 132]. The drug can effectively reduce the formation of cell matrix around the tumor tissue by targeting TGF-β and anti-PD-L1 to further promote the penetration of T cells into the center of the tumor and trigger a more effective anti-tumor immune effect [131–133]. It also has fewer adverse effects on the tumor microenvironment and is safer compared with pan-TGF-β blocking. In this review, we illuminate that the specific blocking of TGF-β1 is a good way to avoid the uncertainty caused by the indiscriminate suppression of TGF-β signal pathways. However, we still face great challenges in the future. For example, how to design and screen drugs targeting TGF-β1 and how to further reduce or eliminate the side effects of targeted drugs. All of these problems require more in-depth thinking and more effective solution strategies.
